# Methyl 8-bromo-3-[1-(4-meth­oxy­phen­yl)-4-oxo-3-phenyl­azetidin-2-yl]-1-methyl-1,2,3,3a,4,9b-hexa­hydro­chromeno[4,3-*b*]pyrrole-3a-carboxyl­ate

**DOI:** 10.1107/S1600536813024963

**Published:** 2013-09-21

**Authors:** P. Sharmila, G. Jagadeesan, Rajesh Raju, Raghunathan Raghavachary, S. Aravindhan

**Affiliations:** a Department of Physics, Presidency College, Chennai 600 005, India; b Department of Organic Chemistry, University of Madras, Guindy Campus, Chennai 600 025, India

## Abstract

In the title compound, C_30_H_29_BrN_2_O_5_, the β-lactam ring is essentially planar, with the O atom displaced from this plane by 0.856 (9) Å, and forming dihedral angles of 24.35 (13) and 89.42 (14)° with the planes of the benzene substituent groups on this ring. The tetra­hydro­pyran ring adopts an envelope conformation with the C atom bearing the β-lactam ring as the flap. In the crystal, weak C—H⋯O hydrogen bonds with carboxyl and tetra­hydro­pyran O-atom acceptors give rise to a chain structure extending along the *b*-axis direction.

## Related literature

For general background to β-lactams, see: Brakhage (1998 ▶). For a related structure, see: Sundaramoorthy *et al.* (2012 ▶). For conformation of the mol­ecular structure, see: Nardelli (1983 ▶).
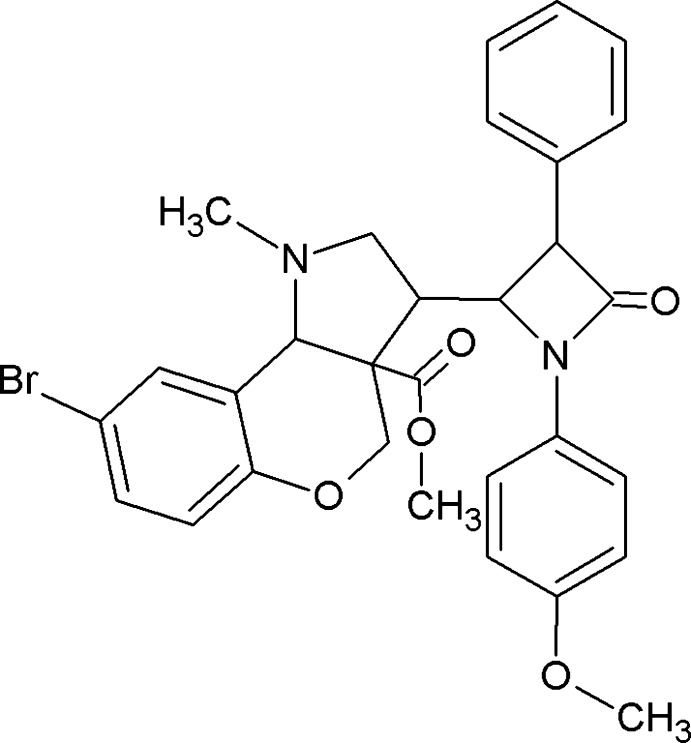


## Experimental

### 

#### Crystal data

C_30_H_29_BrN_2_O_5_
*M* *_r_* = 577.46
Monoclinic, 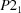

*a* = 10.904 (5) Å
*b* = 10.765 (5) Å
*c* = 11.405 (5) Å
β = 91.681 (5)°
*V* = 1338.2 (11) Å^3^
*Z* = 2
Mo *K*α radiation
μ = 1.58 mm^−1^
*T* = 293 K
0.25 × 0.20 × 0.20 mm

#### Data collection

Bruker Kappa APEXII CCD diffractometer
Absorption correction: multi-scan (*SADABS*; Bruker 2008 ▶) *T*_min_ = 0.979, *T*_max_ = 0.983
28413 measured reflections
6511 independent reflections
5355 reflections with *I* > 2σ(*I*)
*R*_int_ = 0.037

#### Refinement

*R*[*F* ^2^ > 2σ(*F* ^2^)] = 0.030
*wR*(*F* ^2^) = 0.065
*S* = 0.96
6511 reflections
343 parameters
1 restraint
H-atom parameters constrained
Δρ_max_ = 0.42 e Å^−3^
Δρ_min_ = −0.21 e Å^−3^
Absolute structure: Flack (1983 ▶), 2910 Friedel pairs
Absolute structure parameter: 0.006 (5)

### 

Data collection: *APEX2* (Bruker, 2008 ▶); cell refinement: *APEX2* and *SAINT* (Bruker, 2008 ▶); data reduction: *SAINT* and *XPREP* (Bruker, 2008 ▶); program(s) used to solve structure: *SHELXS97* (Sheldrick, 2008 ▶); program(s) used to refine structure: *SHELXL97* (Sheldrick, 2008 ▶); molecular graphics: *ORTEP-3 for Windows* (Farrugia, 2012 ▶); software used to prepare material for publication: *PLATON* (Spek, 2009 ▶).

## Supplementary Material

Crystal structure: contains datablock(s) global, I. DOI: 10.1107/S1600536813024963/zs2268sup1.cif

Structure factors: contains datablock(s) I. DOI: 10.1107/S1600536813024963/zs2268Isup2.hkl

Supplementary material file. DOI: 10.1107/S1600536813024963/zs2268Isup3.cml

Additional supplementary materials:  crystallographic information; 3D view; checkCIF report

## Figures and Tables

**Table 1:** Hydrogen-bond geometry (Å, °)

*D*—H⋯*A*	*D*—H	H⋯*A*	*D*⋯*A*	*D*—H⋯*A*
C20—H20*B*⋯O4^i^	0.97	2.43	3.366 (3)	161
C14—H14⋯O5^ii^	0.93	2.56	3.309 (3)	138

Symmetry codes: (i) 
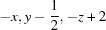
; (ii) 
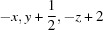
.

**Fig. 1.**
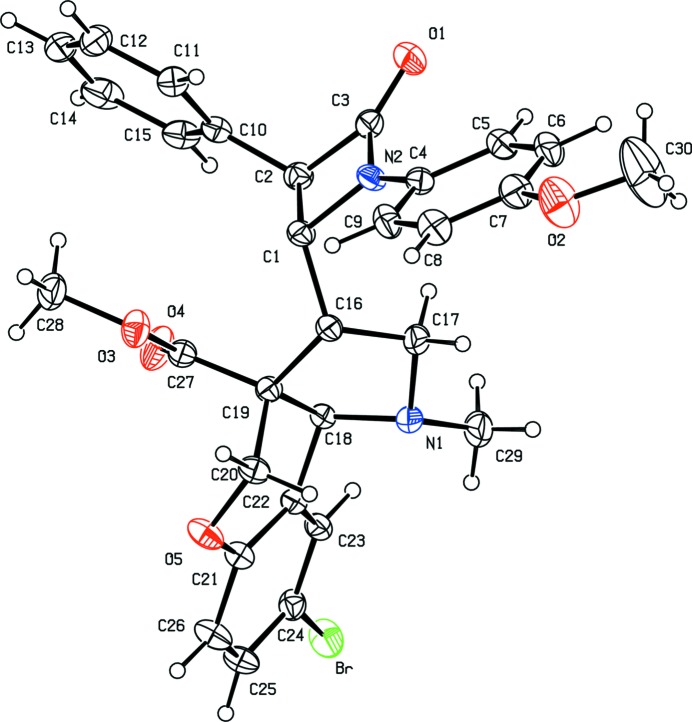
The molecular configuration and atom numbering for the title compound, with displacement ellipsoids drawn at the 30% probability level.

**Fig. 2.**
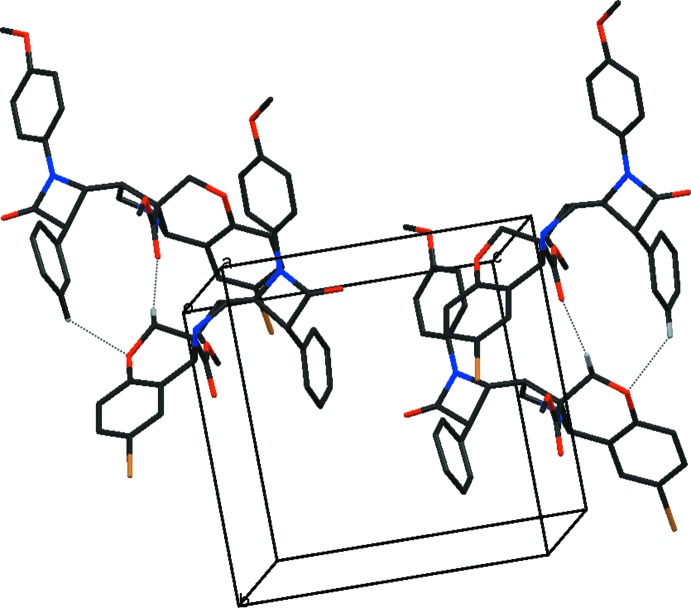
The crystal packing of the title compound, with hydrogen bonds shown as dashed lines. H-atoms not involved in interactions have been omitted.

## supplementary crystallographic information

### 1. Comment

The most commonly used β-lactam antibiotics for the therapy of infectious diseases are penicillin and cephalosporin (Brakhage, 1998). In view of potential applications, the crystal structure determination of the title β-lactam derivative, C_30_H_29_BrN_2_O_5_, was carried out and is reported herein. In this compound (Fig. 1), the four-membered ring of the β-lactam fragment (N2/C1–C3) is essentially planar (r.m.s. deviation = 0.0122 Å), with O1 displaced from the plane by 0.856 (9) Å. The mean-planes of the benzene rings of the two benzene substituent groups defined by C4–C9 and C10–C15 are inclined at dihedral angles 24.35 (13)° and 89.42 (14) °, respectively, with respect to four-membered β-lactam ring. The pyrrolidine ring (N1/C16/C17/C18/C19) adopts an envelope conformation, defined by the asymmetry parameters (Nardelli, 1983), DS (N2) = 0.0789 (19) Å and D2 (C16) = 0.0228 (15) Å whereas the pyran ring [(O5/C18/C19/C20/C21/C22) adopts a C19-envelope/sofa conformation with C19 displaced by 0.385 (1) Å from the least-squares planes formed by the remaining ring atoms.

In the crystal, the structure is stabilized by weak intermolecular aromatic C14—*H*···O5_tetrahydropyran_ and tetrahydropyran C20—*H*···O4_carboxyl_ hydrogen-bonding interactions (Table 1), giving a one-dimensional chain extending along *b* (Fig. 2). Present also in the structure are two intramolecular C—H···β-lactam interactions involving aromatic C11—H11···*Cg* [2.932 (4) Å; C—H···*Cg*, angle 94°] and piperidine C17—H17A···*Cg* [2.842 (4) Å; C—H···*Cg* angle, 100°].

### 2. Experimental

A solution of (*Z*)-methyl 2-[(4-bromo-2-formylphenoxy)methyl]-3-[1-(4-methoxyphenyl)- 4-oxo-3-phenylazetidin-2-yl]acrylate (1 mmol) and sarcosine (1.2 mmol) was refluxed in dry acetonitrile. Completion of the reaction was confirmed by TLC analysis. The solvent was then removed under vacuum, then diluted with dichloromethane and washed with brine and water. The organic layer was separated and removed and the residue was subjected to column chromatography using ethyl acetate and hexane (2:8) as an eluent, affording the cycloadduct. The product was dissolved in ethyl acetate and the resulting solution was allowed to slowly evaporate over 48 h, resulting in the formation of crystals suitable for the single-crystal X-ray diffraction analysis.

### 3. Refinement

Hydrogen atoms were included at calculated positions with C—H ranging from 0.93 to 0.97 Å and treated using a riding model with displacement parameters *U*_iso_(H) = 1.2*U*_eq_(C)(methine, methylene and aromatic), or 1.5*U*_eq_(C)(methyl). The absolute configuration for the molecule was not determined definitively but the local configuration for the five chiral centres [C1(*R*),C2(*R*), C16(*S*),C18(*R*),C19(*R*) for the trivial atom numbering scheme used] was assigned on the basis of the Flack parameter [0.006 (5) for 2910 Friedel pairs].

### Crystal data

**Table:**

C_30_H_29_BrN_2_O_5_	*F*(000) = 596
*M**_r_* = 577.46	*D*_x_ = 1.433 Mg m^−^^3^
Monoclinic, *P*2_1_	Mo *K*α radiation, λ = 0.71073 Å
Hall symbol: P 2yb	Cell parameters from 8834 reflections
*a* = 10.904 (5) Å	θ = 2.1–31.2°
*b* = 10.765 (5) Å	µ = 1.58 mm^−^^1^
*c* = 11.405 (5) Å	*T* = 293 K
β = 91.681 (5)°	Block, colourless
*V* = 1338.2 (11) Å^3^	0.25 × 0.20 × 0.20 mm
*Z* = 2	

### Data collection

**Table:**

Bruker Kappa APEXII CCD diffractometer	6511 independent reflections
Radiation source: fine-focus sealed tube	5355 reflections with *I* > 2σ(*I*)
Graphite monochromator	*R*_int_ = 0.037
ω and φ scans	θ_max_ = 28.6°, θ_min_ = 2.6°
Absorption correction: multi-scan (*SADABS*; Bruker 2008)	*h* = −14→14
*T*_min_ = 0.979, *T*_max_ = 0.983	*k* = −12→14
28413 measured reflections	*l* = −15→15

### Refinement

**Table:**

Refinement on *F*^2^	Secondary atom site location: difference Fourier map
Least-squares matrix: full	Hydrogen site location: inferred from neighbouring sites
*R*[*F*^2^ > 2σ(*F*^2^)] = 0.030	H-atom parameters constrained
*wR*(*F*^2^) = 0.065	*w* = 1/[σ^2^(*F*_o_^2^) + (0.0292*P*)^2^] where *P* = (*F*_o_^2^ + 2*F*_c_^2^)/3
*S* = 0.96	(Δ/σ)_max_ = 0.001
6511 reflections	Δρ_max_ = 0.42 e Å^−^^3^
343 parameters	Δρ_min_ = −0.21 e Å^−^^3^
1 restraint	Absolute structure: Flack (1983), 2910 Friedel pairs
Primary atom site location: structure-invariant direct methods	Absolute structure parameter: 0.006 (5)

### Special details

**Table:**

Geometry. All e.s.d.'s (except the e.s.d. in the dihedral angle between two l.s. planes) are estimated using the full covariance matrix. The cell e.s.d.'s are taken into account individually in the estimation of e.s.d.'s in distances, angles and torsion angles; correlations between e.s.d.'s in cell parameters are only used when they are defined by crystal symmetry. An approximate (isotropic) treatment of cell e.s.d.'s is used for estimating e.s.d.'s involving l.s. planes.
Refinement. Refinement of *F*^2^ against ALL reflections. The weighted *R*-factor *wR* and goodness of fit *S* are based on *F*^2^, conventional *R*-factors *R* are based on *F*, with *F* set to zero for negative *F*^2^. The threshold expression of *F*^2^ > σ(*F*^2^) is used only for calculating *R*-factors(gt) *etc*. and is not relevant to the choice of reflections for refinement. *R*-factors based on *F*^2^ are statistically about twice as large as those based on *F*, and *R*- factors based on ALL data will be even larger.

### Fractional atomic coordinates and isotropic or equivalent isotropic displacement parameters (Å^2^)

**Table:**

	*x*	*y*	*z*	*U*_iso_*/*U*_eq_	
Br	0.400982 (17)	0.56044 (2)	0.676638 (19)	0.05395 (8)	
O1	0.31783 (11)	0.04829 (18)	1.46057 (11)	0.0508 (4)	
O3	−0.04275 (11)	0.10982 (13)	1.05918 (12)	0.0453 (4)	
C18	0.26814 (15)	0.19721 (18)	0.96769 (15)	0.0300 (4)	
H18	0.2783	0.2600	1.0295	0.036*	
N2	0.24186 (14)	−0.05496 (15)	1.29243 (13)	0.0346 (4)	
C19	0.15975 (16)	0.11200 (16)	0.99504 (15)	0.0288 (4)	
C17	0.35296 (16)	0.0409 (2)	1.08186 (17)	0.0460 (5)	
H17A	0.3798	0.0894	1.1495	0.055*	
H17B	0.3987	−0.0364	1.0816	0.055*	
C11	−0.00677 (18)	0.12700 (19)	1.39886 (16)	0.0389 (4)	
H11	0.0019	0.0414	1.4062	0.047*	
O5	0.08439 (12)	0.12441 (13)	0.79140 (12)	0.0415 (3)	
C1	0.16563 (17)	0.01715 (17)	1.20853 (15)	0.0322 (4)	
H1	0.0796	−0.0092	1.2090	0.039*	
C10	0.08118 (17)	0.19488 (18)	1.34185 (15)	0.0334 (4)	
C20	0.12485 (17)	0.04159 (18)	0.88268 (15)	0.0352 (4)	
H20A	0.1953	−0.0050	0.8569	0.042*	
H20B	0.0599	−0.0171	0.8986	0.042*	
N1	0.37101 (13)	0.11007 (16)	0.97373 (13)	0.0408 (4)	
C23	0.32095 (15)	0.36424 (19)	0.82176 (15)	0.0342 (4)	
H23	0.3774	0.3968	0.8762	0.041*	
C2	0.18887 (17)	0.13190 (18)	1.28871 (17)	0.0349 (4)	
H2	0.2427	0.1922	1.2515	0.042*	
O4	0.04486 (13)	0.29512 (13)	1.04029 (15)	0.0551 (4)	
C7	0.34735 (18)	−0.4280 (2)	1.28407 (17)	0.0469 (5)	
C21	0.16422 (17)	0.21901 (17)	0.76857 (16)	0.0332 (4)	
C27	0.04967 (16)	0.18462 (18)	1.03411 (15)	0.0321 (4)	
C8	0.2427 (2)	−0.3891 (2)	1.2253 (2)	0.0476 (5)	
H8	0.1955	−0.4460	1.1823	0.057*	
C22	0.25184 (15)	0.25992 (17)	0.85049 (15)	0.0299 (4)	
C4	0.27709 (16)	−0.18125 (18)	1.29142 (15)	0.0340 (4)	
C13	−0.1218 (2)	0.3116 (3)	1.43409 (19)	0.0557 (6)	
H13	−0.1902	0.3504	1.4642	0.067*	
C3	0.26291 (15)	0.0416 (2)	1.36740 (15)	0.0367 (4)	
C9	0.20708 (18)	−0.2667 (2)	1.22956 (18)	0.0415 (5)	
H9	0.1353	−0.2416	1.1904	0.050*	
C14	−0.0354 (2)	0.3795 (2)	1.37900 (18)	0.0562 (6)	
H14	−0.0446	0.4651	1.3718	0.067*	
O2	0.37564 (17)	−0.55173 (16)	1.27246 (18)	0.0755 (5)	
C16	0.21506 (16)	0.01548 (18)	1.08484 (16)	0.0348 (4)	
H16	0.2005	−0.0677	1.0523	0.042*	
C25	0.22241 (19)	0.3750 (2)	0.63222 (17)	0.0424 (5)	
H25	0.2147	0.4121	0.5587	0.051*	
C15	0.0665 (2)	0.3214 (2)	1.33349 (17)	0.0453 (5)	
H15	0.1256	0.3688	1.2969	0.054*	
C26	0.1497 (2)	0.2749 (2)	0.66016 (17)	0.0456 (5)	
H26	0.0911	0.2453	0.6062	0.055*	
C12	−0.10739 (19)	0.1852 (2)	1.44493 (18)	0.0488 (5)	
H12	−0.1657	0.1387	1.4835	0.059*	
C6	0.4169 (2)	−0.3436 (2)	1.34782 (19)	0.0480 (5)	
H6	0.4871	−0.3699	1.3889	0.058*	
C5	0.38299 (18)	−0.2206 (2)	1.35108 (18)	0.0414 (5)	
H5	0.4310	−0.1637	1.3932	0.050*	
C24	0.30595 (17)	0.41881 (18)	0.71383 (17)	0.0363 (4)	
C30	0.4857 (4)	−0.5944 (3)	1.3249 (4)	0.1324 (18)	
H30A	0.4940	−0.6819	1.3109	0.199*	
H30B	0.4850	−0.5793	1.4078	0.199*	
H30C	0.5535	−0.5512	1.2918	0.199*	
C28	−0.15281 (18)	0.1671 (2)	1.1007 (2)	0.0540 (6)	
H28A	−0.2127	0.1041	1.1157	0.081*	
H28B	−0.1850	0.2235	1.0423	0.081*	
H28C	−0.1340	0.2119	1.1718	0.081*	
C29	0.49131 (19)	0.1676 (3)	0.9721 (2)	0.0615 (7)	
H29A	0.4995	0.2127	0.9002	0.092*	
H29B	0.5534	0.1045	0.9780	0.092*	
H29C	0.5006	0.2237	1.0372	0.092*	

### Atomic displacement parameters (Å^2^)

**Table:**

	*U*^11^	*U*^22^	*U*^33^	*U*^12^	*U*^13^	*U*^23^
Br	0.04262 (11)	0.05682 (14)	0.06218 (14)	−0.01288 (11)	−0.00226 (8)	0.02366 (12)
O1	0.0458 (7)	0.0643 (11)	0.0415 (7)	0.0033 (9)	−0.0118 (6)	−0.0051 (8)
O3	0.0307 (7)	0.0432 (8)	0.0623 (9)	−0.0010 (6)	0.0053 (6)	0.0079 (7)
C18	0.0282 (9)	0.0361 (10)	0.0254 (9)	0.0008 (7)	−0.0022 (7)	0.0006 (7)
N2	0.0394 (9)	0.0372 (9)	0.0269 (8)	0.0017 (7)	−0.0042 (7)	0.0046 (7)
C19	0.0321 (9)	0.0270 (8)	0.0273 (9)	0.0027 (7)	0.0005 (7)	0.0026 (7)
C17	0.0363 (9)	0.0596 (16)	0.0423 (10)	0.0153 (10)	0.0049 (8)	0.0145 (11)
C11	0.0426 (11)	0.0362 (10)	0.0378 (11)	−0.0026 (9)	0.0016 (9)	−0.0025 (8)
O5	0.0512 (8)	0.0370 (8)	0.0354 (7)	−0.0124 (6)	−0.0145 (6)	0.0052 (6)
C1	0.0332 (9)	0.0333 (10)	0.0299 (9)	0.0024 (7)	−0.0002 (7)	0.0061 (7)
C10	0.0394 (10)	0.0340 (10)	0.0264 (9)	−0.0004 (8)	−0.0040 (8)	−0.0015 (7)
C20	0.0440 (10)	0.0283 (11)	0.0330 (9)	−0.0001 (8)	−0.0034 (7)	0.0024 (8)
N1	0.0316 (8)	0.0588 (11)	0.0324 (8)	0.0110 (7)	0.0052 (7)	0.0111 (7)
C23	0.0284 (9)	0.0430 (11)	0.0309 (9)	−0.0042 (8)	−0.0035 (7)	0.0012 (8)
C2	0.0336 (10)	0.0371 (11)	0.0340 (10)	−0.0033 (8)	0.0022 (8)	0.0023 (8)
O4	0.0498 (9)	0.0317 (9)	0.0850 (12)	0.0077 (6)	0.0228 (8)	0.0021 (8)
C7	0.0531 (11)	0.0383 (11)	0.0492 (11)	0.0043 (12)	0.0001 (9)	0.0079 (11)
C21	0.0370 (10)	0.0316 (10)	0.0309 (10)	−0.0014 (8)	−0.0029 (8)	0.0007 (8)
C27	0.0327 (10)	0.0354 (11)	0.0282 (9)	0.0014 (8)	0.0008 (7)	0.0053 (8)
C8	0.0491 (12)	0.0418 (12)	0.0516 (13)	−0.0047 (9)	−0.0031 (10)	0.0038 (10)
C22	0.0288 (9)	0.0337 (10)	0.0272 (9)	0.0016 (7)	−0.0005 (7)	−0.0010 (7)
C4	0.0354 (10)	0.0376 (11)	0.0291 (9)	0.0013 (8)	0.0027 (8)	0.0094 (8)
C13	0.0561 (14)	0.0730 (18)	0.0377 (12)	0.0260 (12)	−0.0030 (10)	−0.0154 (12)
C3	0.0309 (8)	0.0443 (13)	0.0350 (9)	−0.0008 (9)	0.0035 (7)	0.0034 (9)
C9	0.0370 (10)	0.0392 (11)	0.0478 (12)	−0.0001 (9)	−0.0059 (9)	0.0113 (9)
C14	0.0914 (18)	0.0418 (13)	0.0351 (11)	0.0229 (12)	−0.0043 (12)	−0.0045 (10)
O2	0.0852 (13)	0.0409 (10)	0.0990 (15)	0.0164 (9)	−0.0241 (11)	−0.0022 (10)
C16	0.0384 (10)	0.0344 (10)	0.0318 (9)	0.0091 (7)	0.0022 (8)	0.0023 (7)
C25	0.0528 (12)	0.0413 (11)	0.0325 (10)	0.0003 (9)	−0.0065 (9)	0.0095 (9)
C15	0.0664 (13)	0.0359 (11)	0.0336 (10)	0.0012 (10)	0.0012 (10)	0.0011 (8)
C26	0.0568 (13)	0.0434 (12)	0.0357 (11)	−0.0075 (10)	−0.0177 (9)	0.0023 (9)
C12	0.0403 (11)	0.0644 (16)	0.0419 (12)	−0.0023 (10)	0.0018 (9)	−0.0107 (11)
C6	0.0474 (12)	0.0491 (13)	0.0470 (12)	0.0131 (10)	−0.0074 (10)	0.0063 (10)
C5	0.0407 (11)	0.0453 (12)	0.0379 (11)	−0.0002 (9)	−0.0040 (8)	0.0042 (9)
C24	0.0318 (10)	0.0358 (11)	0.0415 (11)	−0.0011 (8)	0.0032 (8)	0.0070 (8)
C30	0.150 (4)	0.070 (2)	0.173 (4)	0.060 (2)	−0.077 (3)	−0.026 (3)
C28	0.0317 (11)	0.0705 (16)	0.0601 (14)	0.0056 (10)	0.0089 (10)	0.0124 (12)
C29	0.0323 (11)	0.098 (2)	0.0544 (14)	0.0065 (12)	0.0057 (10)	0.0240 (14)

### Geometric parameters (Å, º)

**Table:**

Br—C24	1.898 (2)	O4—C27	1.193 (2)
O1—C3	1.206 (2)	C7—O2	1.375 (3)
O3—C27	1.328 (2)	C7—C8	1.371 (3)
O3—C28	1.442 (2)	C7—C6	1.377 (3)
C18—N1	1.462 (2)	C21—C26	1.380 (3)
C18—C22	1.503 (2)	C21—C22	1.388 (3)
C18—C19	1.535 (3)	C8—C9	1.375 (3)
C18—H18	0.9800	C8—H8	0.9300
N2—C3	1.361 (3)	C4—C9	1.377 (3)
N2—C4	1.413 (3)	C4—C5	1.389 (3)
N2—C1	1.471 (2)	C13—C14	1.361 (4)
C19—C27	1.511 (3)	C13—C12	1.374 (4)
C19—C20	1.527 (3)	C13—H13	0.9300
C19—C16	1.567 (3)	C9—H9	0.9300
C17—N1	1.459 (2)	C14—C15	1.389 (3)
C17—C16	1.530 (3)	C14—H14	0.9300
C17—H17A	0.9700	O2—C30	1.403 (4)
C17—H17B	0.9700	C16—H16	0.9800
C11—C12	1.381 (3)	C25—C24	1.367 (3)
C11—C10	1.383 (3)	C25—C26	1.381 (3)
C11—H11	0.9300	C25—H25	0.9300
O5—C21	1.370 (2)	C15—H15	0.9300
O5—C20	1.431 (2)	C26—H26	0.9300
C1—C16	1.525 (3)	C12—H12	0.9300
C1—C2	1.553 (3)	C6—C5	1.376 (3)
C1—H1	0.9800	C6—H6	0.9300
C10—C15	1.375 (3)	C5—H5	0.9300
C10—C2	1.499 (3)	C30—H30A	0.9600
C20—H20A	0.9700	C30—H30B	0.9600
C20—H20B	0.9700	C30—H30C	0.9600
N1—C29	1.451 (3)	C28—H28A	0.9600
C23—C24	1.369 (3)	C28—H28B	0.9600
C23—C22	1.397 (3)	C28—H28C	0.9600
C23—H23	0.9300	C29—H29A	0.9600
C2—C3	1.536 (3)	C29—H29B	0.9600
C2—H2	0.9800	C29—H29C	0.9600
			
C27—O3—C28	117.11 (17)	C21—C22—C23	117.56 (16)
N1—C18—C22	113.60 (14)	C21—C22—C18	121.13 (16)
N1—C18—C19	101.61 (15)	C23—C22—C18	121.27 (15)
C22—C18—C19	112.16 (14)	C9—C4—C5	119.19 (19)
N1—C18—H18	109.7	C9—C4—N2	120.04 (17)
C22—C18—H18	109.7	C5—C4—N2	120.76 (18)
C19—C18—H18	109.7	C14—C13—C12	119.6 (2)
C3—N2—C4	134.35 (16)	C14—C13—H13	120.2
C3—N2—C1	95.01 (15)	C12—C13—H13	120.2
C4—N2—C1	130.64 (16)	O1—C3—N2	132.0 (2)
C27—C19—C20	108.89 (15)	O1—C3—C2	135.7 (2)
C27—C19—C18	111.95 (15)	N2—C3—C2	92.30 (15)
C20—C19—C18	107.48 (14)	C8—C9—C4	120.37 (19)
C27—C19—C16	116.38 (14)	C8—C9—H9	119.8
C20—C19—C16	107.51 (15)	C4—C9—H9	119.8
C18—C19—C16	104.19 (14)	C13—C14—C15	120.2 (2)
N1—C17—C16	105.50 (15)	C13—C14—H14	119.9
N1—C17—H17A	110.6	C15—C14—H14	119.9
C16—C17—H17A	110.6	C7—O2—C30	117.9 (2)
N1—C17—H17B	110.6	C1—C16—C17	113.12 (16)
C16—C17—H17B	110.6	C1—C16—C19	117.20 (15)
H17A—C17—H17B	108.8	C17—C16—C19	103.09 (15)
C12—C11—C10	120.6 (2)	C1—C16—H16	107.7
C12—C11—H11	119.7	C17—C16—H16	107.7
C10—C11—H11	119.7	C19—C16—H16	107.7
C21—O5—C20	114.68 (14)	C24—C25—C26	119.22 (18)
N2—C1—C16	112.79 (15)	C24—C25—H25	120.4
N2—C1—C2	87.56 (14)	C26—C25—H25	120.4
C16—C1—C2	119.87 (15)	C10—C15—C14	120.8 (2)
N2—C1—H1	111.5	C10—C15—H15	119.6
C16—C1—H1	111.5	C14—C15—H15	119.6
C2—C1—H1	111.5	C25—C26—C21	119.68 (19)
C15—C10—C11	118.39 (19)	C25—C26—H26	120.2
C15—C10—C2	120.73 (18)	C21—C26—H26	120.2
C11—C10—C2	120.88 (18)	C13—C12—C11	120.3 (2)
O5—C20—C19	111.42 (16)	C13—C12—H12	119.8
O5—C20—H20A	109.3	C11—C12—H12	119.8
C19—C20—H20A	109.3	C5—C6—C7	120.3 (2)
O5—C20—H20B	109.3	C5—C6—H6	119.8
C19—C20—H20B	109.3	C7—C6—H6	119.8
H20A—C20—H20B	108.0	C6—C5—C4	120.02 (19)
C29—N1—C17	111.89 (16)	C6—C5—H5	120.0
C29—N1—C18	114.70 (18)	C4—C5—H5	120.0
C17—N1—C18	104.17 (13)	C25—C24—C23	121.57 (18)
C24—C23—C22	120.31 (16)	C25—C24—Br	118.84 (15)
C24—C23—H23	119.8	C23—C24—Br	119.59 (14)
C22—C23—H23	119.8	O2—C30—H30A	109.5
C10—C2—C3	117.03 (15)	O2—C30—H30B	109.5
C10—C2—C1	118.78 (16)	H30A—C30—H30B	109.5
C3—C2—C1	85.10 (15)	O2—C30—H30C	109.5
C10—C2—H2	111.2	H30A—C30—H30C	109.5
C3—C2—H2	111.2	H30B—C30—H30C	109.5
C1—C2—H2	111.2	O3—C28—H28A	109.5
O2—C7—C8	115.7 (2)	O3—C28—H28B	109.5
O2—C7—C6	124.6 (2)	H28A—C28—H28B	109.5
C8—C7—C6	119.6 (2)	O3—C28—H28C	109.5
O5—C21—C26	115.94 (16)	H28A—C28—H28C	109.5
O5—C21—C22	122.46 (16)	H28B—C28—H28C	109.5
C26—C21—C22	121.56 (17)	N1—C29—H29A	109.5
O4—C27—O3	123.86 (17)	N1—C29—H29B	109.5
O4—C27—C19	124.76 (17)	H29A—C29—H29B	109.5
O3—C27—C19	111.37 (17)	N1—C29—H29C	109.5
C7—C8—C9	120.4 (2)	H29A—C29—H29C	109.5
C7—C8—H8	119.8	H29B—C29—H29C	109.5
C9—C8—H8	119.8		
			
N1—C18—C19—C27	160.79 (14)	N1—C18—C22—C23	−82.7 (2)
C22—C18—C19—C27	−77.54 (19)	C19—C18—C22—C23	162.77 (16)
N1—C18—C19—C20	−79.68 (16)	C3—N2—C4—C9	155.5 (2)
C22—C18—C19—C20	41.99 (19)	C1—N2—C4—C9	−23.8 (3)
N1—C18—C19—C16	34.21 (16)	C3—N2—C4—C5	−25.4 (3)
C22—C18—C19—C16	155.88 (14)	C1—N2—C4—C5	155.29 (18)
C3—N2—C1—C16	122.88 (16)	C4—N2—C3—O1	−2.0 (3)
C4—N2—C1—C16	−57.6 (2)	C1—N2—C3—O1	177.5 (2)
C3—N2—C1—C2	1.36 (14)	C4—N2—C3—C2	179.15 (19)
C4—N2—C1—C2	−179.14 (18)	C1—N2—C3—C2	−1.38 (14)
C12—C11—C10—C15	0.7 (3)	C10—C2—C3—O1	−57.5 (3)
C12—C11—C10—C2	−178.30 (19)	C1—C2—C3—O1	−177.5 (2)
C21—O5—C20—C19	53.2 (2)	C10—C2—C3—N2	121.22 (17)
C27—C19—C20—O5	58.75 (18)	C1—C2—C3—N2	1.30 (13)
C18—C19—C20—O5	−62.71 (18)	C7—C8—C9—C4	1.0 (3)
C16—C19—C20—O5	−174.35 (13)	C5—C4—C9—C8	−1.1 (3)
C16—C17—N1—C29	165.70 (18)	N2—C4—C9—C8	178.07 (18)
C16—C17—N1—C18	41.2 (2)	C12—C13—C14—C15	0.3 (3)
C22—C18—N1—C29	69.9 (2)	C8—C7—O2—C30	176.1 (3)
C19—C18—N1—C29	−169.48 (16)	C6—C7—O2—C30	−2.9 (4)
C22—C18—N1—C17	−167.51 (16)	N2—C1—C16—C17	−46.8 (2)
C19—C18—N1—C17	−46.84 (18)	C2—C1—C16—C17	54.0 (2)
C15—C10—C2—C3	130.1 (2)	N2—C1—C16—C19	−166.55 (15)
C11—C10—C2—C3	−50.9 (3)	C2—C1—C16—C19	−65.7 (2)
C15—C10—C2—C1	−130.03 (19)	N1—C17—C16—C1	−145.59 (17)
C11—C10—C2—C1	49.0 (2)	N1—C17—C16—C19	−18.0 (2)
N2—C1—C2—C10	−119.46 (17)	C27—C19—C16—C1	−8.8 (2)
C16—C1—C2—C10	125.54 (18)	C20—C19—C16—C1	−131.17 (17)
N2—C1—C2—C3	−1.21 (12)	C18—C19—C16—C1	114.96 (17)
C16—C1—C2—C3	−116.21 (17)	C27—C19—C16—C17	−133.75 (17)
C20—O5—C21—C26	159.63 (18)	C20—C19—C16—C17	103.88 (17)
C20—O5—C21—C22	−22.6 (2)	C18—C19—C16—C17	−9.99 (18)
C28—O3—C27—O4	−2.6 (3)	C11—C10—C15—C14	−1.3 (3)
C28—O3—C27—C19	178.39 (16)	C2—C10—C15—C14	177.70 (18)
C20—C19—C27—O4	−116.7 (2)	C13—C14—C15—C10	0.8 (3)
C18—C19—C27—O4	2.0 (3)	C24—C25—C26—C21	−1.5 (3)
C16—C19—C27—O4	121.6 (2)	O5—C21—C26—C25	176.88 (19)
C20—C19—C27—O3	62.25 (19)	C22—C21—C26—C25	−0.9 (3)
C18—C19—C27—O3	−179.05 (15)	C14—C13—C12—C11	−0.9 (3)
C16—C19—C27—O3	−59.4 (2)	C10—C11—C12—C13	0.4 (3)
O2—C7—C8—C9	−178.98 (19)	O2—C7—C6—C5	177.9 (2)
C6—C7—C8—C9	0.1 (3)	C8—C7—C6—C5	−1.1 (3)
O5—C21—C22—C23	−174.34 (16)	C7—C6—C5—C4	1.0 (3)
C26—C21—C22—C23	3.3 (3)	C9—C4—C5—C6	0.1 (3)
O5—C21—C22—C18	3.3 (3)	N2—C4—C5—C6	−179.07 (18)
C26—C21—C22—C18	−179.12 (19)	C26—C25—C24—C23	1.4 (3)
C24—C23—C22—C21	−3.4 (3)	C26—C25—C24—Br	−177.57 (16)
C24—C23—C22—C18	179.01 (17)	C22—C23—C24—C25	1.1 (3)
N1—C18—C22—C21	99.8 (2)	C22—C23—C24—Br	−179.94 (14)
C19—C18—C22—C21	−14.8 (2)		

### Hydrogen-bond geometry (Å, º)

**Table:**

*D*—H···*A*	*D*—H	H···*A*	*D*···*A*	*D*—H···*A*
C20—H20*B*···O4^i^	0.97	2.43	3.366 (3)	161
C14—H14···O5^ii^	0.93	2.56	3.309 (3)	138

Symmetry codes: (i) −*x*, *y*−1/2, −*z*+2; (ii) −*x*, *y*+1/2, −*z*+2.
